# Correction: Validating self-reported exclusive breastfeeding in Eswatini using stable isotope techniques

**DOI:** 10.1371/journal.pone.0350654

**Published:** 2026-05-29

**Authors:** Kwanele Siyabonga Simelane, Tholakele Mhlanga, Nhlakanipho Sandziso Mkhaliphi, Siniketiwe Zwane, Glorious Dlamini, Henry Gadaga, Helen Mulol, Anna Coutsoudis

The Funding statement for this article is incorrect. The correct Funding statement is as follows: The authors would like to acknowledge the International Atomic Energy Agency (IAEA) for providing technical expertise and resources in supporting the Ministry of Health to undertake the study through Technical Cooperation Project SWA6003.
