# Supplementary material for: Validating self-reported exclusive breastfeeding in Eswatini using stable isotope techniques
Source: PLoS One. 2026 Apr 15;21(4):e0346799. doi: 10.1371/journal.pone.0346799 (PMC13082701; doi:10.1371/journal.pone.0346799)
Supplement: Attachment — Submitted filename: Responses to Reviewer.docx [file pone.0346799.s002.docx]

**General Comment.**

You have indicated that data is available from “ehhrrbeswatini@gmail.com.” Please can we ask you to provide us with a general contact email address for the data requests, so readers can request access in perpetuity. If a general email is not available, please provide a link to a website where readers can obtain access to data.

**Response**

The general contact email address for data requests, through which readers can obtain access on an ongoing basis, is “es@ehhrrb.org.sz”. This address is managed by the Eswatini Health and Human Research Review Board (EHHRRB), the national health research ethics committee under the Eswatini Ministry of Health. It is actively monitored by the Board’s administrator.

**Responses to Reviewer’s Comments**

**Reviewer #1**

1. ***Comment***

*Line 19: the EBF data collected using the 24-hour recall presented is a prevalence (i.e. a snapshot of the percent of infants who are exclusively breastfed at one point in time), not a rate. Suggest reframing throughout the paper as the "prevalence of exclusive breastfeeding".*

**Response**

Thank you for your comment. We have changed this throughout the paper as suggested. Example below:

**Original Text**

Exclusive breastfeeding (EBF) rates at the population level are typically assessed through maternal or caregiver reports, which are prone to recall and social desirability biases.

**Revised text**

Exclusive breastfeeding (EBF) prevalence at the population level is typically assessed through maternal or caregiver reports, which are prone to recall and social desirability biases.

2. *Comment*

*Lines 31-34: In the abstract, referring to the models as Model 1 and Model 2 is not sufficient for readers to understand the difference. Suggest clarifying that the difference between the two models is the cutoff that was used to determine EBF using the DTM.*

**Response**

We appreciate the valuable comment. Clarity has been provided.

**Original Text**

Using Model 1 cutoffs, EBF rates by maternal recall vs. DTM were 100% vs. 50% (≤2.4 months), 79.5% vs. 18.2% (2.5–4 months), and 71.9% vs. 18.8% (4.1–5.5 months). For Model 2, rates were 100% vs. 65.4%, 79.5% vs. 50%, and 71.9% vs. 43.8%, respectively.

**Revised text**

Using Model 1 cutoffs, prevalences of EBF by maternal recall vs. DTM were 100% vs. 50% (≤2.4 months), 79.5% vs. 18.2% (2.5–4 months), and 71.9% vs. 18.8% (4.1–5.5 months). For Model 2, rates were 100% vs. 65.4%, 79.5% vs. 50%, and 71.9% vs. 43.8%, respectively. The difference between the two models is the cutoff that was used to determine EBF using the DTM method.

3. *Comment;*

*Lines 36-38: The conclusion to strengthen breastfeeding education does not flow logically from the data presented. For one, this is a recommendation, not a conclusion. The data presented suggests that there is a discrepancy between the DTM and the recall method, most likely due to overreporting. In my opinion, if a recommendation were to emerge from this finding, it would be related to improving the measurement of EBF or recommendations about interpreting 24 hour recall EBF data when using it for program monitoring and evaluation.*

**Response**

Thank you for your valuable comments, the conclusion has been revised to align with the findings.

**Original text**

The lower EBF rates detected by DTM highlight the urgent need to strengthen breastfeeding education in Eswatini to combat childhood malnutrition.

**Revised text**

Maternal recall substantially overestimated exclusive breastfeeding prevalence, while the deuterium oxide technique yields more reliable, objective assessments of infant feeding practices, highlighting the need to interpret recall data with caution due to overreporting.

4. *Comment*

*Line 44-46: There is evidence that stunting that occurs before age 2 is potentially reversible under the right conditions. So I checked the reference cited here. This source does not seem to support the sentence. Please cite another source, or rewrite to align with the data in this source.*

**Response**

Thank you for your comment. The source has been revised and the wording of the sentence has been arranged such that “acute malnutrition” comes before the “irreversible stunting”.

**Original Text**

The first two years of life represent a critical window for fostering healthy growth, establishing positive behaviours, and preventing irreversible stunting and acute malnutrition, ensuring that children reach their full developmental potential (2).

**Revised text**

The first two years of life represent a critical window for fostering healthy growth, establishing positive behaviours, and preventing acute malnutrition and stunting, ensuring that children reach their full developmental potential (5).

5. *Comment*

*Line 47: Define EBF here.*

**Response**

Thank you. Exclusive breastfeeding has been defined.

**Original text**

The World Health Organization (WHO) recommends that babies be exclusively breastfed for the first six months after which complementary feeding must be introduced (3)

**Revised text**

The World Health Organization (WHO) recommends that babies be exclusively breastfed (i.e., an infant receives only breast milk, with no other liquids or solids, not even water, for the first six months of life, except for oral rehydration solution, vitamin/mineral drops, or medicines) after which complementary feeding must be introduced (6).

6. *Comment: Line 49: Stunting is not a risk factor for poor child development per se, but a marker of poor child development (see LeRoy and Frongillo 2019).*

**Response**

Thank you for the valuable comment, indeed stunting is not necessarily a risk factor but is a marker of poor child development. Therefore, the wording of the sentence has been revised.

**Original text**

Exclusive breastfeeding (EBF) is a major protective factor against stunting, which is a well-established risk for poor child development (4–6).

**Revised text**

Exclusive breastfeeding (EBF) is a major protective factor against stunting, which is a well-established marker for poor child development (7-12).

7. *Comment*

*Lines 51-53: Since this manuscript is solely about EBF, suggest removing references to other IYCF monitoring.*

**Respnse**

IYCF monitoring information has been removed.

**Original text**

It is therefore imperative for countries to routinely collect and interpret Infant and Young Child Feeding (IYCF) data to monitor malnutrition trends toward country specific and global targets and develop evidence-based policies and programs to address feeding practices. EBF is one of critical data elements for IYCF.

**Revised text**

It is therefore imperative for countries to routinely collect and interpret infant feeding and growth indicator data to monitor malnutrition trends toward country specific and global targets and develop evidence-based policies and programs to address feeding practices. EBF is one of the critical data elements for infant feeding.

8. *Comment*

*Lines 53-54: Since EBF here refers to the prevalence estimate, suggest revising to read "EBF prevalence among 0-6 month olds" to be more precise.*

**Response**

Thank you. We agree with your suggestion, and the wording has been revised.

**Original text**

As of 2022 in Eswatini, EBF rates during the first six months measured through maternal recall assessments were reported at 77.28%, while stunting among children under five years remained significant at 25% (7,8).

**Revised text**

As of 2022 in Eswatini, the prevalence of EBF 0-6 months old, measured through maternal recall assessments, was reported at 77.28%, while stunting among children under five years remained significant at 25% (13,14).

9. *Comment*

*Lines 68: The manuscript did not mention any policies in Eswatini nor how these findings could be used to inform or change policies. The data presented in this manuscript seems to assess the validity of EBF measured using the recall method; or to improve understanding of differences in EBF measurement by comparing recall and DTM. The purpose/goal of the study should be restated.*

**Response**

Thank you very much for this valuable comment. We have restated the study’s purpose to clarify that the main objective was to assess the validity of EBF measurement methods and to provide evidence that could inform improvements in national monitoring and policy strategies.

**Original text**

The goal is to generate more accurate data on breastfeeding practices to inform policy.

**Revised text**

The main objective is to assess the validity of EBF measurement methods and to provide evidence that could inform improvements in national monitoring and policy strategies.

10. *Comments*

*Line 74: Please clarify whether this is the national prevalence of poverty or whether this is referring to rural poverty. It is unclear as stated. Also clarify whether the 54.8% refers to people or households.*

**Response**

The World Bank “poverty headcount ratio at national poverty lines (% of population)” for Eswatini is defined as the percentage of the population living below the national poverty line.

World Bank poverty headcount indicators are consistently person‑based (population share), even though they are calculated from household survey data. We have revised the text to address your questions.

**Original Text**

About 75.6% of the population live in rural areas (15), with a poverty rate of 54.8% (16).

**Revised text**

Nationally, 54.8% of the population lives below the national poverty line, with poverty concentrated in rural areas where about 70% of rural residents are poor (21). Approximately 75.6% of the population resides in rural areas.

11. *Comment*

*Lines71-79: Suggest moving to background section.*

**Response**

The text has been moved as suggested.

12. *Comment*

*Lines 102-130: This section combines the sample collection and processing with the analysis of the samples. If the editor agrees, suggest separating these two and describing the laboratory analysis of the saliva sample in the analysis section.*

**Response**

Thank you for your comment. Sample collection is now separated from sample analysis.

13. *Comment*

*Lines 134-135: The authors state that, "Model 2 was developed based on 24-hour observations of mothers practicing EBF." However, the study methods do not include observations, so this is confusing. I believe Model 2 only differs from Model 1 in that different cutoffs for NMOI were used to determine exclusive, predominant or partial BF. Also, please add a reference and clarify where the 86.6 cutoff came from.*

**Response**

Thank you for your comment. We agree that our original statement could be misinterpreted. Model 2 does not involve direct observations from our dataset; rather, the NMOI cutoff applied in this model was derived from a previous study (Liu et al., 2019) that included observed mother–infant pairs practicing exclusive breastfeeding in Indonesia. This cutoff (86.6) corresponds to the 90th percentile of the posterior distribution of NMOI in that population. We have revised the text to clarify this and added the Liu et al. study as a new reference (now reference 23). Model 2 differs from Model 1 only in the use of this empirically derived cutoff to classify exclusive breastfeeding status.

Reference added:

23. Liu Z, Diana A, Slater C, Preston T, Gibson RS, Houghton L, Duffull SB. Development of a nonlinear hierarchical model to describe the disposition of deuterium in mother–infant pairs to assess exclusive breastfeeding practice. J Pharmacokinet Pharmacodyn. 2019;46(1):1–13. [https://doi.org/10.1007/s10928-018-9613-x*Comment*](https://doi.org/10.1007/s10928-018-9613-xComment)

14. *Lines 138-145: The description of the DTM sample collection and analysis is very detailed. However, there are few details about how the 24 hour recall data were collected. Where were mothers interviewed? When were they interviewed (at the beginning or at the end of the 14 day period)? How was EBF determined? Was it according to the WHO guidance, exactly? Authors state that the WHO recommended questionnaire was modified, but do not say how, which makes me wonder whether the analysis was modified as well.*

**Response**

Thank you for your valuable comment. We have revised this section to clear all the grey areas. Below is the revised paragraph.

**Original Text**

Maternal recall was assessed using a questionnaire on infant feeding practices. Breastfeeding indicators were obtained through interviews with mothers, using a questionnaire adapted from the WHO/UNICEF Indicators for Assessing Infant and Young Child Feeding, Part 2 (18). The tool is based on current guidelines regarding breastfeeding, complementary feeding, and the feeding of non-breastfed infants and young children under two years of age (18). It is divided into three modules: a household roster, a breastfeeding initiation module (BF), and an infant and young child feeding (IYCF) module. Modified versions of this questionnaire have been used in several breastfeeding studies (10–13).

**Revised text**

Maternal recall was assessed using a questionnaire on infant feeding practices. Through an interview, mothers reported infant feeding practices over the previous 24 hours, detailing breast milk intake alongside any liquids, semi-solids, or solids given. Breastfeeding indicators were obtained through interviews with mothers, using a questionnaire adapted from the WHO/UNICEF Indicators for Assessing Infant and Young Child Feeding, Part 2 (24) at the onset of sample collection, just before participants received health services at the facility. The tool is based on current guidelines regarding breastfeeding, complementary feeding, and the feeding of non-breastfed infants and young children under two years of age (25). It is divided into three modules: a household roster, a breastfeeding initiation module (BF), and an infant and young child feeding (IYCF) module. Modified versions of this questionnaire have been used in several breastfeeding studies (16–19) and have adapted the standard WHO "recall since birth" method for local cultural contexts, local practices, and also to confine it within the limits of feeding infants less than the age of six months. EBF was determined by maternal reports confirming no non-breast milk foods/liquids since birth or in the prior 24 hours. This aligned closely with WHO indicators (no liquids/solids beyond breast milk, medicines, or oral rehydration up to the reference age, 6 months), though adaptations addressed local practices.

15. *Comment*

*Lines 156-158: This needs a reference.*

**Response;** We have provided a reference.

Reference cited: McHugh ML. Interrater reliability: the kappa statistic. Biochem Medica. 2012;22(3):276–82.

16. *Comment*

*Line 163: Please clarify what "up to high school education" means. Does this mean that 74.5% reported high school as the highest level of education completed? Does it mean 74.5% reported at least some high school education? Does this mean that 74.5% completed high school or less?*

**Response**

In this case up to high school education means reached and/or completed high school. Some reached but did not complete high school. Others reported that they have reached and completed high school. So, the 74.5% is some or completed high school combined into one group.

**Original text**

The majority of mothers had up to high school education (74.5%), were unemployed (86.3%) or single (56.9%), as shown in….

**Revised text**

The majority of mothers reported some or completed high school as the highest level of education (74.5%), were unemployed (86.3%) or single (56.9%), as shown in Table 1.

17. *Comment*

*Figure 1, Model 1: I am surprised to see that EBF was the same in the middle and older age group. I would have expected more of a difference. Have other studies had similar findings?*

**Response**

No, most studies report declining exclusive breastfeeding (EBF) prevalences as infants age from younger (e.g., 0-2 months) to middle/older groups (e.g., 4-6 months) within the 0-6 month period, contrary to the similar EBF prevalences observed in Model 1 (Figure 1). Declining EBF prevalences were, however, found for the less stringent cut-off, which was the more expected trend, and this further supports the conclusion that Model 2 more accurately reflects true EBF than Model 1.

18. *Comment*

*Line 198: On line 156, the authors state that a score of -1.0 is "perfect disagreement" and that 0.21-0.40 [positive] represent fair agreement. So, I am confused that these negative kappa coefficients are described as "fair agreement" instead of fair disagreement. Please reconcile.*

**Response**

Thank you for raising this key comment. We have corrected the kappa interpretation of the results.

**Original Text**

Models 1 and 2 gave Kappa values of –0.25 and -0.27 respectively, as shown in Table 2. These Kappa values represent only a fair agreement.

**Revised text**

Models 1 and 2 gave Kappa values of –0.25 and -0.27 respectively, as shown in Table 2. These Kappa values represent a fair disagreement.

19. *Comment*

*Lines 213-216: This paragraph does not seem very relevant. Suggest deleting or revise to make the relevance to your findings more explicit.*

**Response**

Your comment is greatly appreciated. We have revised the text to make it relevant to the findings.

**Original text**

The majority of women in the study had attained only secondary education (74.5%) and had not progressed to tertiary-level education. Furthermore, a substantial proportion (86.3%) were unemployed. Comparable socio-demographic profiles in other regions of East and Southeast Asia have been associated with significant adverse effects on infant feeding practices.

**Revised text**

In this study, most participants had only secondary education (74.5%) and were unemployed (86.3%). These socio-demographic characteristics may have contributed to the low prevalence of exclusive breastfeeding observed, as limited education and unemployment can restrict access to accurate breastfeeding information, reduce engagement with maternal health services, and affect women’s confidence or autonomy in infant feeding decisions. Similar patterns have been reported in studies from East and Southeast Asia, where lower education and unemployment were significantly associated with reduced exclusive breastfeeding prevalences

20. *Comment*

*Lines 219-220: Do the methods allow you to distinguish between consumption of supplementary foods, other milks, beverages, and water? Could the non-breast milk consumption be any of these things? I am not sure why it only mentions foods. Unless data from the survey suggests it is food and not water or other milks.*

**Response**

DTM methods primarily quantify breast milk intake and any non-milk oral intake (NMOI), which encompasses water, other milks, beverages, supplementary foods, or their combinations, rather than distinguishing these precisely. We have revised the paragraph to reflect the other NMOI.

**Original text**

These findings showed that some infants were introduced to supplementary foods when they were as young as less than 3 months, indicating poor adherence to EBF for the first 6 months as recommended by the WHO.

**Revised Text**

These findings showed that some infants were introduced to non-milk oral intake (NMOI), which is water intake from water, other milks, beverages, supplementary foods, or their combinations, before the age of 3 months, indicating poor adherence to the WHO recommendation of EBF for the first 6 months.

21. *Comment*

*Line 240: Social desirability bias likely results in the overestimation. However, it was difficult to assess how much it might have played a role here. For example, the mother-infant dyads were recruited from health centers, so presumably all of them had been exposed to breastfeeding promotion messages. This may increase this bias. Other factors like who interviewed them and when are not mentioned, making it difficult to assess. Finally, were any steps taken to reduce social desirability bias? For example, introductory language before the questions, asking questions in different ways, etc. There is not enough detail in the methods section about how the questionnaire was administered to get a good grasp of this.*

**Response**

Effort was done to minimize social desirability bias including establishing rapport, interviewer behavior, and de‑emphasis of evaluation

In interviews or supervised data collection, focused on trust, neutrality, and non‑judgment.

Interviewers were trained to;

- Maintain neutral facial expressions and tone, and avoid praise like “good answer.”
- Read questions verbatim and avoid suggesting what most people say.
- Quickly normalize sensitive behaviors (“A lot of people tell me they sometimes do this”).
- Use opening small‑talk and non‑threatening questions to build rapport before sensitive items.
- Emphasize that services, are not affected by responses:

“Your decision to participate and the answers you give will not affect the care you receive or your relationship with this clinic.” This point was part of the consent form they signed before the start of the interview.

- The most sensitive questions were placed later in the instrument, after some trust and engagement are established**.**

The primary potential source of social desirability bias in this study was unavoidable, as participant recruitment took place at the health facility where they routinely received training and counseling on the benefits of exclusive breastfeeding.

22. *Comment*

*Lines 243-252: It is worth mentioning that the 24 hour recall is just one day of the 14 observed using the DTM (assuming the interview occurred within the 14 day period, which is not stated in the methods). So complete agreement is not expected if mothers do not feed their infants in the same way every single day. It is also worth discussing how the 24 hour method is a proxy, not a direct measure, of EBF for the duration of the first six months as recommended by WHO, though it is often misinterpreted as such.*

**Response**

Thank you for your valuable comment, the information on when the interview occurred is addressed in the revised version of the data collection section. We indicated that it was done at the inception of the data collection. This was also part of screening process because mothers who explicitly declare not exclusively breastfeeding or not breastfeeding at all, were excluded from the research. Those who were exclusively breastfeeding and intend to do so for the whole first six months of the baby’s life, were included in the study.

23. *Comment*

*Lines 254-260: The conclusions are not supported by the findings. Suggest rewriting this section completely.*

**Response:**

Thank you for your comment. Conclusion has been revised.

**Original text**

This study indicates that the maternal recall method when used alone may not be sufficient to estimate the EBF rates in a country. Hence there is a need to strengthen breastfeeding education among mothers to reduce the rate of childhood malnutrition in Eswatini. The DTM can be used to validate self-reported data, especially when working with smaller sample sizes. The study findings support the widely accepted global recommendation for frequent monitoring of health data including breastfeeding data to effectively inform policies and interventions aimed at improving child health and nutrition.

**Revised text**

This study validates the deuterium oxide dose-to-mother (DTM) technique as the biochemical gold standard for assessing exclusive breastfeeding (EBF) against maternal recall in Eswatini. Maternal recall substantially overestimated EBF prevalence across infant age groups, revealing inconsistencies due to recall inaccuracies and social desirability bias. These findings confirm DTM's superiority for validation studies despite measuring breastfeeding over a short observation period. Integrating DTM enhances methodological rigor in breastfeeding research.

**Reviewer #2:**

1. *General comment*

*Thanks for the opportunity to review this paper, which compares EBF as reported using the WHO IYCF recall method and a stable isotope technique. It is helpful to have objective measures of EBF to better understand the reliability of the recall method. I think this is an important paper, but my overarching comment is about the framing. It’s not clear why this needs to be framed as important for child stunting. There are many other and more well documented reasons EBF is important for the health of infants. I would suggest framing the paper (in the intro and discussion) so that it focuses on the importance of accurate measurement of EBF and the potential influence of social desirability on survey responses. The discussion could talk more about what low agreement between the recall and isotope methods means for policy and practice. Countries are making policies based on the prevalence of EBF from Demographic and Health Surveys, Multiple Indicator Cluster Surveys, or SMART surveys. What should they do now, if the survey data on EBF are not accurate?*

**Response**

Thank you for the valuable comment. Our opening statements highlight the multiple benefits of exclusive breastfeeding (EBF). When EBF is not practiced, the infant loses an essential opportunity for optimal early-life nutrition and immune protection, leaving the child more vulnerable to repeated health challenges during critical growth periods. The EBF period serves as a crucial window to mitigate the effects of malnutrition that may have occurred in utero and to reduce the risk of subsequent episodes of malnutrition linked to socioeconomic and environmental factors. By providing complete nutrition and immune support, EBF directly addresses several key drivers of poor child health outcomes. Stunting, a marker of impaired child growth and development, often results from recurrent episodes of malnutrition caused by inadequate dietary intake or nutrient loss due to illness—conditions that breastfeeding helps protect against.

In addition to my overarching comments, I would like to offer the following specific comments to the authors:

2. *Comment*

*Lines 44-45 – I suggest either removing the word “irreversible” or switching the order of “irreversible stunting” and “acute malnutrition.” The current order makes it seem like acute malnutrition is also irreversible, which is not true.*

**Response**

Thank you for your valuable comment. Switching it makes sense. We have revised the sentence as shown below.

**Original text**

The first two years of life represent a critical window for fostering healthy growth, establishing positive behaviours, and preventing irreversible stunting and acute malnutrition, ensuring that children reach their full developmental potential.

**Revised Text**

The first two years of life represent a critical window for fostering healthy growth, establishing positive behaviours, and preventing acute malnutrition and stunting, ensuring that children reach their full developmental potential.

3. *Comment*

*Lines 48-50 – There is not much evidence that EBF is a protective factor against stunting. The Victora et al. paper cited does not show that EBF is related to stunting reduction. I suggest revising this sentence to highlight the documented benefits of EBF (as outlined in the Victora paper).*

**Response**

Thank you for your observation. We have revised the references, the Victoria paper indeed does not show evidence that EBF is related to stunting reduction, but a recent paper by Laksmi et al., 2025, p. 30. It concludes that exclusive breastfeeding for babies during the first six months of life is essential to meet nutritional needs and increase their body’s immunity, reducing the risk of stunting. In addition, Putri et a., 2024, also support this by concluding that exclusive breastfeeding affects the stunting in toddlers.

4. *Comments*

*Lines 50-52 – Please revise this sentence. IYCF indicators are not used to monitor malnutrition trends. Growth indicators (WHZ, HAZ, WAZ) are used for monitoring malnutrition trends.*

**Response**

Thank you for your valuable comment, IYCF information has been removed.

**Original text**

It is therefore imperative for countries to routinely collect and interpret Infant and Young Child Feeding (IYCF) data to monitor malnutrition trends toward country specific and global targets and develop evidence-based policies and programs to address feeding practices. EBF is one of critical data elements for IYCF.

**Revised text**

It is therefore imperative for countries to routinely collect and interpret Infant feeding and growth indicator data to monitor malnutrition trends toward country specific and global targets. This data is valuable to inform the development of evidence-based policies and programs which address feeding practices in particular the protection, promotion and support of EBF.

5. First paragraph of Background section – It’s not clear why the authors are emphasizing the connection between EBF and stunting in the first paragraph of the Background section. It seems like it would make sense to focus in this paragraph on the well-established benefits of EBF.

**Response**

Thank you for this helpful comment. We agree that the opening paragraph should first establish the well-documented benefits of exclusive breastfeeding (EBF) before addressing its association with stunting. We have therefore revised the paragraph to emphasize the established health and developmental benefits of EBF, setting a clearer foundation for the later discussion on its relationship to stunting.

**Revised text**

Exclusive breastfeeding (EBF) for the first 6 months of life is widely recognized as a cornerstone of optimal infant feeding, providing all required nutrients and fluids while protecting against gastrointestinal and respiratory infections and related mortality [1]. EBF also offers important protection against common infectious diseases such as diarrhoea and respiratory infections and may reduce the risk of overweight and obesity later in life [2]. In addition, longer and more exclusive breastfeeding has been associated with improved neurocognitive outcomes, including higher verbal intelligence and better verbal and spatial skills [3] and confers health benefits for mothers, such as lower risks of breast and ovarian cancer [4].

6. Line 92 – How did you determine if the mothers were “clinically well”?

**Response**

Thank you for seeking for clarity. “Clinically well” was determined by mothers having no acute illness and no known chronic or obstetric conditions that could compromise safe participation in the DTM procedure or normal breastfeeding at the time of enrollment.

**Original text**

The study eligibility criteria included clinically well mothers aged 15–49 years

**Revised text**

The study eligibility criteria included mothers aged 15–49 years who were clinically well (no acute/chronic illness affecting breastfeeding).

7. Lines 139-140 – Please explain what kind of adaptations were made to the WHO IYCF questionnaire.

**Response**

Thank you for your comment. The WHO IYCF questionnaire was adapted primarily to ensure cultural and contextual relevance while maintaining alignment with the original indicators. Specifically, the standard “recall since birth” method was modified to reflect local infant-feeding practices and to focus on infants below six months of age. The questions were phrased to capture maternal reports confirming exclusive breastfeeding—defined as no intake of non-breast milk foods or liquids since birth or in the preceding 24 hours. These modifications preserved the integrity of WHO’s definitions (no other liquids or solids, except medicines or oral rehydration salts) while ensuring that the terminology and examples were appropriate and understandable within the local cultural context.

8. Methods – Please include a sentence in the methods section stating that you collected data on the mothers’ socioeconomic characteristics. Please add a subsection on your sample size calculation and how you decided on the number of infants to include in each age group. In addition, it would be helpful to describe the sampling process. Was it purely convenience sampling or did you do stratified sampling by infant age?

**Response**

Thank you for your valuable feedback. We have now added a sentence in the Methods section indicating that data were collected on mothers’ socioeconomic characteristics. We have also included a subsection detailing our sample size determination and the categorization of infants by age. The revised text clarifies that sampling was conducted by convenience from high-volume postnatal care facilities, and that infant age categorization was performed during data analysis in order to separate EBF into 3 categories which reflected early EBF to later EBF with similar sample sizes. Specifically, 130 mother–baby pairs were recruited, of which 102 met the inclusion criteria (such as infants aged 6 weeks to 5.5 months).

9. Table 1 – What do the numbers at the far-right side of the table represent? If they are not important, I would suggest removing them.

**Response**

The numbers at the far-right side of Table 1 are the line numbers which the journal requires and were automatically inserted on all lines, including the Table. They are therefore not part of the Table and can be ignored.

10. Section starting on line 188 – Please add sentences to this section for the other two age groups. The text in this section currently only describes results for the youngest age group.

**Revised text:**

Among infants aged ≤2.4 months, maternal recall indicated an EBF prevalence of 100%, as shown in Fig 2. In contrast, when assessed objectively using the DTM method, the EBF prevalence was substantially lower: 50% based on Model 1 (NMOI ≤ 25 g/day) and 65.4% based on Model 2 (NMOI ≤ 86.6 g/day). Among infants aged 2.5–4.0 months, EBF prevalence decreased further, with maternal recall reporting 80%, whereas DTM-based estimates showed only 22.7% using Model 1 and 50% using Model 2. Similarly, in the 4.1–5.5-month age group, maternal recall indicated an EBF prevalence of 70%, but DTM assessment revealed markedly lower values—approximately 21.9% using Model 1 and 43.8% based on Model 2.

11. Table 1 – I suggest using the same number of decimal places throughout the table. Two decimal places should be sufficient.

**Response**

The percentages were kept with one decimal place. As per comment 9, the numbers on the right in the Table are line numbers.

12. Line 204 – Please be more specific with your statement here by including what the first study is about.

**Response**

Thank you for this helpful suggestion. We have revised the statement to explicitly describe the study's focus on exclusive breastfeeding practices/rates, clarifying that no prior Eswatini-based research has applied the DTM method to this topic.

**Original text**

To our knowledge, this is the first study to be reported in Eswatini.

**Revised text**

To the best of our knowledge, this is the first study in Eswatini to investigate exclusive breastfeeding using the DTM method.

13. Lines 209-210 – It’s not clear what issue related to measuring EBF the authors are referring to here. Please add more information about the issues mentioned.

**Response**

The sentence has been revised to remove the unnecessary phrase “the issue”.

**Original text**

The issue of measuring EBF has been explored extensively in both past and recent scholarly work (10,12,21–25)

**Revised text**

Measuring EBF has been explored extensively in both past and recent scholarly work [16,18,27–31].

14. Lines 213-216 – It’s not clear how this is relevant to your main findings. Depending on the context, women with higher levels of education are less likely to EBF than women with lower levels. So, is your point that these women are not well enough educated or too well educated?

**Response**

Your comment is greatly appreciated. We have revised the text to make it relevant to the findings.

**Original text**

The majority of women in the study had attained only secondary education (74.5%) and had not progressed to tertiary-level education. Furthermore, a substantial proportion (86.3%) were unemployed. Comparable socio-demographic profiles in other regions of East and Southeast Asia have been associated with significant adverse effects on infant feeding practices.

**Revised text**

In this study, most participants had only secondary education (74.5%) and were unemployed (86.3%). These socio-demographic characteristics may have contributed to the low prevalence of exclusive breastfeeding observed, as limited education and unemployment can restrict access to accurate breastfeeding information, reduce engagement with maternal health services, and affect women’s confidence or autonomy in infant feeding decisions. Similar patterns have been reported in studies from East and Southeast Asia, where lower education and unemployment were significantly associated with reduced exclusive breastfeeding prevalences.

15. Line 236 – I would suggest replacing “addressing child malnutrition” with “ensuring optimal infant feeding practices.”

**Response**

Thank you for the valuable suggestion. I agree that “ensuring optimal infant feeding practices” provides a clearer and more precise expression of the intended focus. The recommended change has been accepted and incorporated into the revised version.

**Original text**

Currently policy interventions are based on maternal recall and may not have been effective in addressing child malnutrition.

**Revised text**

Currently policy interventions are based on maternal recall and may not have been effective in ensuring optimal infant feeding practices.

16. Line 241 – You may want to add a sentence about social desirability in the measurement of EBF in relation to this study by Christine Stewart et al., 2025: <https://www.sciencedirect.com/science/article/pii/S247529912401713X>

**Response**

Thank you for highlighting this important study by Stewart et al. (2025); it compellingly demonstrates social desirability bias in self-reported exclusive breastfeeding (EBF), where 75.9% of intervention mothers revised earlier reports of early cessation to claim ≥6 months EBF, versus 32.5% in controls, likely due to recall distortion from nutrition messaging. This reinforces the need for objective biomarkers like deuterium dose-to-mother methods in trials to validate EBF beyond maternal recall.

We have added some text and cited the reference by Stewart et al as follows:

In the study by Stewart et al.[36], social desirability bias significantly influenced self-reported EBF outcomes in a Kenyan randomized trial promoting infant feeding practices. Researchers observed that mothers in the nutrition intervention group, exposed to breastfeeding promotion, frequently altered their reports over repeated surveys—75.9% shifted from reporting EBF cessation before 6 months to 6 months or later, compared to only 32.5% in the non-nutrition group—suggesting recall bias rather than true behavioral change. This highlights the need for objective EBF measures, like deuterium dose-to-mother techniques, to mitigate such reporting distortions in intervention studies.

17. Line 244-246 - This is the first time a stakeholder participatory approach is mentioned. If you are going to talk about this as a strength, please mention it in the methods section, although I don’t understand how a participatory approach is relevant as a strength in this case.

**Response**

“Thank you for this helpful observation. In our context, the ‘stakeholder participatory approach’ referred to the active involvement of Ministry of Health counterparts and facility managers in site selection, study procedures, and feedback of results, to ensure that the findings were aligned with national information needs and could be readily used for program decision‑making. To address your concern, we have now:

Added a brief description of this participatory involvement in the Methods (Study design) to make it explicit.

Rephrased the statement in the Discussion to clarify that this approach strengthened the policy relevance and local applicability of the findings, rather than implying a methodological advantage for the measurement of EBF itself.

**Revised text**

Methods – Study design section

At national level, the study was implemented in collaboration with the Eswatini National Nutrition Council and senior Ministry of Health officials, who contributed to selection of study sites, refinement of data‑collection procedures and tools, and agreement on how results would be reported back to program stakeholders. This participatory process was intended to enhance the relevance and uptake of the findings for breastfeeding and child‑nutrition policies.

Discussion- Strength paragraph line

Secondly, close collaboration with national and facility‑level stakeholders throughout site selection, implementation and feedback of results is likely to have increased the relevance and applicability of the findings for Eswatini’s breastfeeding and child‑nutrition programs, although it did not affect the objective measurement of EBF itself.

18. Lines 255-256 – I suggest removing the sentence “Hence there is a need to strengthen breastfeeding education among mothers to reduce the rate of childhood malnutrition in Eswatini.” This conclusion does not follow from your results. In fact, it is likely that the breastfeeding education women are already receiving is contributing to the socially desirable responses they are giving on the WHO IYCF questionnaire.

**Response**

Thank you for this insightful comment. We agree that the earlier conclusion overstated the study’s implications and could be misinterpreted as prescriptive. In response, we have removed the sentence suggesting the need to strengthen breastfeeding education. The revised conclusion now focuses on the methodological validation of the deuterium oxide dose-to-mother (DTM) technique, emphasizing its superiority over maternal recall for assessing exclusive breastfeeding in Eswatini. This revision aligns the conclusion more directly with our study’s findings.

**Original text**

This study indicates that the maternal recall method when used alone may not be sufficient to estimate the EBF rates in a country. Hence there is a need to strengthen breastfeeding education among mothers to reduce the rate of childhood malnutrition in Eswatini. The DTM can be used to validate self-reported data, especially when working with smaller sample sizes. The study findings support the widely accepted global recommendation for frequent monitoring of health data including breastfeeding data to effectively inform policies and interventions aimed at improving child health and nutrition**.**

**Revised Text**

This study validates the deuterium oxide dose-to-mother (DTM) technique as the biochemical gold standard for assessing exclusive breastfeeding (EBF) against maternal recall in Eswatini. Maternal recall substantially overestimated EBF prevalence across infant age groups, revealing inconsistencies due to recall inaccuracies and social desirability bias. These findings confirm DTM's superiority for validation studies, despite measuring breastfeeding over a short observation period. Integrating DTM enhances methodological rigor in breastfeeding research.
